# Supplementation with N-3 Long-Chain Polyunsaturated Fatty Acids or Olive Oil in Men and Women with Renal Disease Induces Differential Changes in the DNA Methylation of FADS2 and ELOVL5 in Peripheral Blood Mononuclear Cells

**DOI:** 10.1371/journal.pone.0109896

**Published:** 2014-10-17

**Authors:** Samuel P. Hoile, Rebecca Clarke-Harris, Rae-Chi Huang, Philip C. Calder, Trevor A. Mori, Lawrence J. Beilin, Karen A. Lillycrop, Graham C. Burdge

**Affiliations:** 1 Academic Unit of Human Development and Health, Faculty of Medicine, University of Southampton, Southampton, United Kingdom; 2 Telethon Kids Institute, Perth, Australia; 3 NIHR Southampton Biomedical Research Centre, University Hospital Southampton NHS Foundation Trust and University of Southampton, Southampton, United Kingdom; 4 School of Medicine and Pharmacology, Royal Perth Hospital Unit, University of Western Australia, Perth, Australia; 5 Centre for Biological Sciences, Faculty of Natural and Environmental Sciences, University of Southampton, Southampton, United Kingdom; Max Delbrueck Center for Molecular Medicine, Germany

## Abstract

**Background:**

Studies in animal models and in cultured cells have shown that fatty acids can induce alterations in the DNA methylation of specific genes. There have been no studies of the effects of fatty acid supplementation on the epigenetic regulation of genes in adult humans.

**Methods and Results:**

We investigated the effect of supplementing renal patients with 4 g daily of either n-3 long-chain polyunsaturated fatty acids (n-3 LCPUFA) or olive oil (OO) for 8 weeks on the methylation status of individual CpG loci in the 5′ regulatory region of genes involved in PUFA biosynthesis in peripheral blood mononuclear cells from men and women (aged 53 to 63 years). OO and n-3 LCPUFA each altered (>10% difference in methylation) 2/22 fatty acid desaturase (FADS)-2 CpGs, while n-3 LCPUFA, but not OO, altered (>10%) 1/12 ELOVL5 CpGs in men. OO altered (>6%) 8/22 FADS2 CpGs and (>3%) 3/12 elongase (ELOVL)-5 CpGs, while n-3 LCPUFA altered (>5%) 3/22 FADS2 CpGs and 2/12 (>3%) ELOVL5 CpGs in women. FADS1 or ELOVL2 methylation was unchanged. The n-3 PUFA supplementation findings were replicated in blood DNA from healthy adults (aged 23 to 30 years). The methylation status of the altered CpGs in FADS2 and ELOVL5 was associated negatively with the level of their transcripts.

**Conclusions:**

These findings show that modest fatty acid supplementation can induce altered methylation of specific CpG loci in adult humans, contingent on the nature of the supplement and on sex. This has implications for understanding the effect of fatty acids on PUFA metabolism and cell function.

## Introduction

Epigenetics refers to a group of heterogeneous, but interrelated processes that regulate transcription without changing the DNA nucleotide sequence. Specifically, epigenetics involves methylation at the 5′ position of cytosine bases in CpG dinucleotide pairs, covalent modifications of histones, or the activities of non-coding RNA species [1]. Although the DNA methylation of some gene promoters, for example those involved in cell differentiation or imprinting, is induced in early life and persists throughout the life course, other DNA methylation marks appear to be more plastic [2], particularly during periods of rapid growth [3]. Altered epigenetic regulation by DNA methylation of specific genes has been implicated as a causal factor in a number of non-communicable diseases [4], [5]. Genes that retain epigenetic plasticity may respond to environmental inputs, including nutrition, and so may alter gene and cell function. Thus understanding the impact of nutrient intakes on the epigenome has important implications for dietary choices in relation to health.

Dietary fat intake can modify capacity for polyunsaturated fatty acid (PUFA) by changing the mRNA expression of Δ6 (D6d) and Δ5 (D5d) desaturases [6], [7]. Although product inhibition is likely to be involved, the underlying mechanism has not been well-described. Changes in dietary fatty acid intake can alter the activity of the PUFA biosynthesis pathway in rodent models via changes in the epigenetic regulation of key genes. Increasing dietary α-linolenic acid content during pregnancy and lactation in mice increased the average methylation of the fatty acid desaturase (Fads)-2, which encodes D6d, promoter and of intron 1 by up to 2% in the liver of dams [8], [9]. Feeding pregnant rats diets containing different amounts of saturated or n-3 long-chain polyunsaturated fatty acids (n-3 LCPUFA) induced hypermethylation of specific CpG loci in the Fads2 promoter and decreased mRNA expression in the liver of the adult offspring. This was accompanied by decreased proportions of docosahexaenoic acid (DHA) and arachidonic acid in membrane and plasma phospholipids [10]. Feeding adult female rats a fish oil-enriched diet for 9 weeks also induced lower Fads2 mRNA expression and increased methylation of specific CpG loci in the Fads2 promoter [10]. These changes were reversed when the animals were switched to a soybean oil-base diet [10]. Feeding dams either 7% and 21% (w/w) safflower oil, hydrogenated soybean oil, butter or fish oil induced increased methylation of specific CpG loci in the Fads2 promoter and decreased its expression in aortae in the adult offspring [11]. Mutation of one CpG locus that was hypermethylated in both the liver and aortae of these offspring, which is located within an estrogen receptor response element, decreased the activity of the Fads2 promoter [11]. This indicates that at least some of the hypermethylated loci were involved directly in the regulation of Fads2 transcription. Together, these findings support the suggestion that variation in the fatty acid supply during development can induce persistent changes in the epigenetic regulation of LCPUFA biosynthesis.

To our knowledge, no study has examined the effects of dietary fatty acid intake on the epigenetic regulation of genes involved in PUFA metabolism in adult humans. In this study, we tested the hypothesis that dietary supplementation with preparations containing either predominately n-9 monounsaturated fatty acids (olive oil; OO) or n-3 LCPUFA induces differential changes in the DNA methylation of genes involved in LCPUFA biosynthesis. We measured methylation status of individual CpG loci in the 5′ regulatory region of FADS2, FADS1 (which encodes D5d) and elongase (ELOVL)-5 and ELOVL2 (which encode elongase 5 and elongase 2, respectively) in DNA extracted from peripheral blood mononuclear cells (PBMCs) isolated from patients with chronic renal disease. We also tested whether any induced changes in DNA methylation were related to the level of mRNA and whether any effects of n-3 LCPUFA on the DNA methylation of genes involved in LCPUFA biosynthesis could be replicated in blood from healthy adults.

## Subjects and Methods

### Ethical statement

The studies were conducted according to the principles expressed in the Declaration of Helsinki. Study 1 was approved by the Royal Perth Hospital Ethics Committee (EC2004/045) and is registered on the Australian Clinical Trials Register (ACTRN012605555588640). Subjects gave informed, written consent. Study 2 received ethical approval from the National Research Ethics Service Committee, South Central – Berkshire (REC reference number 11/SC/0384). Participants provided informed, written consent.

### Dietary intervention trials and sample collection

The samples used in this study were derived from specimens collected in two dietary intervention studies, one in patients with chronic kidney disease at the University of Western Australia (Perth, Australia) (Study 1) and the other in healthy adults at the University of Southampton (Southampton, UK) (Study 2).

The design of the dietary intervention trial in Study 1 has been described in detail elsewhere [12]. The samples used in the current study were from a sub-group of 29 men and women for whom paired before and end of intervention samples were available, who were non-smokers and non-diabetic, with moderate to severe chronic renal impairment (estimated glomerular filtration rate between 15 and 60 ml/min/1.73 m^2^ (normal range≥90 ml/min/1.73 m^2^) and serum creatinine less than 350 µmol/l (normal range 53 to 115 µmol/l). The primary exclusion criteria were use of non-steroidal anti-inflammatory or immunosuppressive medication, consumption of fish oil supplements, consumption of more than one portion of fish per week or consumption of more than 4 alcoholic drinks per day. Six out of the total cohort of 35 subjects only had single samples and so were excluded from the present analysis. The details of the subjects from whom samples were analysed in the current study are summarised in Table 1.

**Table 1:** Subject characteristics at baseline.

	Male		Female	
	Study 1 cohort			
Treatment	n-3 LCPUFA	OO	n-3 LCPUFA	OO
n	8	8	6	7
Age (years)	55±4	61±3	53±6	55±4
BMI (kg/m^2^)	25.7±1.0	27.2±1.4	28.7±2.6	29.5±2.8
eGRF (ml/min/1.73 m^2)^	37.6±4.0	34.9±2.4	36.5±4.0	32.7±3.9
Cholesterol (mmol/l)	5.1±0.2	4.6±0.2	4.8±0.4	4.7±0.2
Triglyceride (mmol/l)	2.4±0.3	1.7±0.2	1.1±0.1	1.8±0.5
Creatinine (µmol/l)	209±22	192±16	151±15	163±17
Glucose (mmol/l)	5.0±0.2	5.0±0.2	4.5±0.3	5.1±0.3
HOMA-IR	3.0±0.4	2.4±0.3	2.3±0.8	3.2±1.0
	**Study 2 cohort**			
n	8		12	
Age (years)	23±1		30±4	
BMI (kg/m^2^)	22.6±0.5		21.5±0.5	

Values are mean ± SEM. n-3 LCPUFA, fish oil; OO, olive oil; BMI, body mass index; eGRF, estimated glomerular filtration rate; HOMA-IR, homeostatic model assessment of insulin resistance. Comparisons within male and female subjects by Student’s unpaired t test showed no significant differences (P>0.05) between treatment groups.

Subjects consumed 4 g n-3 LCPUFA, eicosapenatenoic acid (EPA; 1.8 g), dosocapentaenoic acid (DPA; 0.2 g) and DHA (1.5 g) ethyl esters in four 1 g capsules (Omacor, Solvay Pharmaceuticals, Pymble, NSW, Australia) or 4 g olive oil (OO) in four 1 g capsules (Cardinal Health Australia, Braeside, Victoria, Australia) each day for 8 weeks. The study had a randomised double blind design. Venous blood samples were collected in the fasting state and differential blood counts were determined as described [12]. PBMCs were isolated as described [13], stored at −80°C, transported for analysis on dry ice and stored at −80°C until analysed.

The subjects for comparator analyses in Study 2 were the health subjects in a reference group in a study that compared the effect of fish oil supplementation in individuals who were of normal BMI or clinically obese. Subjects men and women of BMI between 18.5 and 25 kg/m^2^, with fasting plasma glucose <5 mmol/l, triacylglycerol <1.0 mmol/l and total cholesterol less than 5 mmol/l who did not consume fish oil or other oil supplements, who did not eat more than one oily fish meal per week, did not have diagnosed diabetes or chronic gastrointestinal problems and were not pregnant or planning to become pregnant within the study period. Subjects were excluded if they used prescribed medicine to control inflammation, to control blood lipids or to control blood pressure. The characteristics of the subjects in Study 2 are detailed in Table 1. A fasting venous blood sample was collected at baseline and an aliquot of whole blood was stored at −20°C. Subjects then received EPAX6000 TG (EPAX, Oslo, Norway) which provided 1.1 g EPA, 0.1 g DPA plus 0.8 g DHA in the form of a triacylglycerol per day for 12 weeks. A second fasting blood sample was collected at the end of the study and an aliquot of whole blood stored at −20°C.

### Isolation of DNA and analysis of DNA methylation

**Figure 1:**
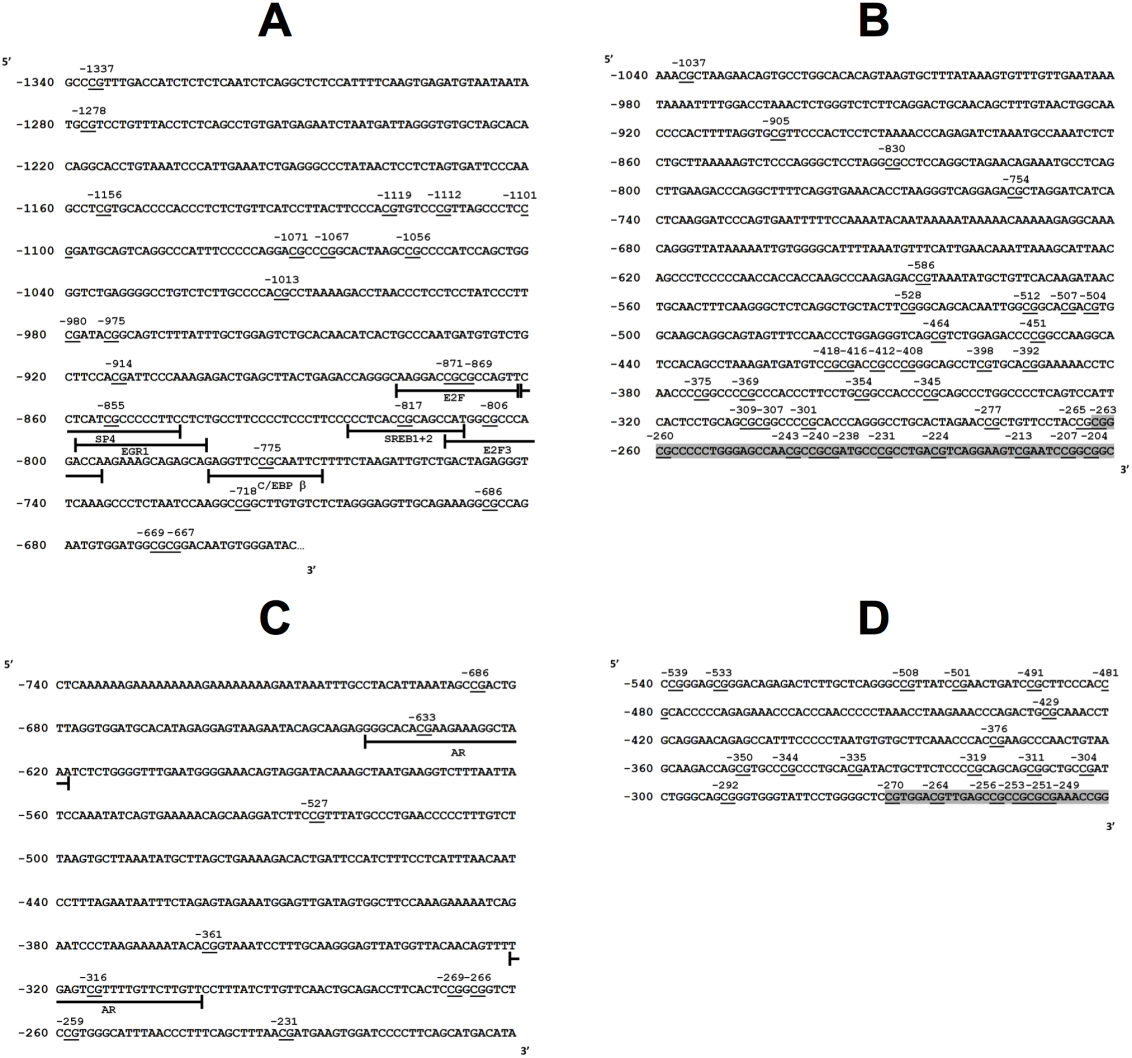
Sequences of the 5′ regions of (A) FADS2, (B) FADS1, (C) ELOVL5 and (D) ELOVL2 that were analysed by pyrosequencing. Individual CpG loci are indicated by their position relative to the transcription start site (bp) and are underlined. Sequences corresponding to CpG islands are indicated grey shading.

Genomic DNA was isolated from 200 µl PBMC or whole blood using the QIAmp DNA blood mini kit (Qiagen, 51106) according to the manufacturer’s instructions. The level of methylation of individual CpG dinucleotides in the 5′ regulatory regions of FADS 1 and 2, and ELOVL 2 and 5 was measured from −504 to −1037, from −18 to −1661, from −292 to −508 and from −6 to −686 bases, respectively, upstream from the transcription start site (Figure 1) using sodium bisulphite pyrosequencing essentially as described [10]. PCR primers and pyrosequencing probes are listed in Tables 2 and 3. Bisulphite conversion was carried out using the EZ DNA methylation kit (ZymoResearch, D5006). Modified DNA was amplified using KAPA2G Robust Hot Start Taq DNA polymerase (Anachem, KK5702). PCR products were immobilised on streptavidin–sepharose beads (GE Healthcare UK Ltd., 17-5113-01), washed, denatured and released into annealing buffer containing the sequencing primers. Pyrosequencing was carried out using CDT reagents (Qiagen, 972824) on a PSQ 96MA machine (Qiagen, 972824). Per cent methylation at each CpG locus was calculated using the Pyro Q CpG software (Biotage). The within assay coefficient of variation was less than 5% for all CpG loci that were measured and the limit of detection was 5% methylation.

**Table 2:** PCR primer sequences.

Primer location(bp relative to TSS)		PCR primer sequences	
Start	End	Forward (5′ to 3′)	Reverse (3′ to 5′)
		FADS1	
–309	–309	GGAGGGTTAGAGTTTGGAGATT	CCCCCCATAAATCTTAAACAACTCACAA
–392	–375	AGTGAATGGATTGAGGGGTTAG	CCCCCACCAAAACATCCACAACCTAA
–504	–418	AGTGAATGGATTGAGGGGTTAG	ACCCCCACCAAAACATCCACA
–586	–528	AGAGGTAAATAGGGTTATAAAAATTGTG	CCTCCAAAATTAAAAACTACTACCTACTTA
–754		AAGAGGTAAATAGGGTTATAAAAATTGTG	CCTCCAAAATTAAAAACTACTACCTACT
–830		AGGGTTTTTAGGAGTTTTTAGGTTAGAA	ACAATTTTTATAACCCTATTTACCTCTTT
–905		AAGAATAGTGTTTGGTATATAGTAAGTG	AACCTCTCCTAACCCTTAAATATTTCACCT
–1037		AAGAATAGTGTTTGGTATATAGTAAGTG	TCTCCTAACCCTTAAATATTTCACCT
		FADS2	
–1661	1655	GTATGGTGGTTTTGAGGATTGTT	AAAATACTCCCTAATTCTACCTTTCAACTA
–1337		TTGTTGTGAAATTTAGATTGGGTAGG	CCTAAAAAAAATAAACCTAACTACAT
–1278		TTGTTGTGAAATTTAGATTGGGTAGG	CCTAAAAAAAATAAACCTAACTACAT
–1156		TTGTTGTGAAATTTAGATTGGGTAGG	CCTAAAAAAAATAAACCTAACTACAT
–1119	–1101	ATTTGAGGGTTTTATAATTTTTTTAGTGAT	ACCCTAATCTCAATAAACTCAATCT
–1071	–1056	ATTTGAGGGTTTTATAATTTTTTTAGTGAT	CCCTAATCTCAATAAACTCAATCTCTT
–1013	–975	ATTTGAGGGTTTTATAATTTTTTTAGTGAT	CCTTACCCTAATCTCAATAAACTCAATC
–914	–855	GGTAGTTTTTATTTGTTGGAGTTTGTAT	AAACCTCTACTCTACTTTCTTAATCT
–817	–775	ATTGAGTTTATTGAGATTAGGGTAAGG	ACTTTAAACCCTCTAATCAAACAATCTT
–718	–667	ATTGTTTGATTAGAGGGTTTAAAGTT	AAACTCCAATATCCCACATTAT
–258	–244	AAGATTTTTTTGGGTTAATGGT	ATCCCTAACTTCCCAATACC
–133	–84	AAGATTTTTTTGGGTTAATGGT	AAATCCCTAACTTCCCAATAC
–64	–50	GGGGAGTTTTTATTGGAGGTAA	AATCCCTAACTTCCCAATACC
–18		TGGGGGTATTGGGAAGTTAG	CCCTCCCCCAACCTTCTC
		ELOVL 2	
–237	–270	TTTGGGTAGAGGGTGGGTATTTT	CCTCTCCCACAAAAACCT
–292	–350	AGATTGAGTAAATTTGTAGGAATAGAGT	AAACCCCAAAAATACCCACC
–376		AGGAGGTTATAGTTTTGTTTATAGTGAAGA	AAACCCCAAAAATACCCACC
–429		AGGAGGTTATAGTTTTGTTTATAGTGAAGA	TCCACCAAACCCCAAAAATACCC
–481	–508	AGGAGGTTATAGTTTTGTTTATAGTGAAGA	CACATTAAAAAAAAATAACTCTATTCCTAC
		ELOVL 5	
–6	–14	GGTTTATTAGGAAGAAAGGGGAAAA	AACCTAAACCCAAATTAACCCC
–59	–100	GGGAGTTATGGTTATAATAGTTTTGAGT	ATTTTTTTCCCCTTTCTTCCTAAT
–134	–185	AGGGAGTTATGGTTATAATAGTTTTGAGT	ACCCTAAACTCTAATTTTTTTACTACCA
–231	–266	TTTTGTAAGGGAGTTATGGTTATAATAGT	ACCCTAAACTCTAATTTTTTTACTACC
–269	–316	AGAGTAGAAATGGAGTTGATAGTGG	ACCCTAAACTCTAATTTTTTTACTACCA
–633	–686	GGAGGTTGTAGTGAGTTGAGA	AACCTTCATTAACTTTATATCCTACTATT

TSS, transcription start site.

**Table 3:** Pyrosequencing primers.

Starting location (bp relative to TSS)	Primer sequence
	FADS1
–260	AGTTTTGGTTTTTTAGTTTATTTA
–309	GATTGAGGGGTTAGG
–392	GGTAGGGGTTGAGGTTT
–504	AGGGTTTTTAGGTTGT
–586	AGTTTTTTTTTAATTATTATTAAGT
–754	AATATTTAAGGGTTAGGAG
–830	CTAAAACATTTCTATTCTAACCT
–905	AGTTTTGTAATTGGTAATTTTA
–1037	GGAGAATTAAATGAGTTAAGG
	FADS2
–1661	TGGTTTTGAGGATTGTTAA
–1337	AGATTGGGTAGGGTT
–1278	GGTTTTTTATTTTTAAGTGAGATG
–1156	GGGTTTTATAATTTTTTTAGTGAT
–1119	AAAATAAACCTAACTACATCC
–1071	CCTCAAACCCCAACT
–1013	ATTATACAAACTCCAACAAAT
–914	ATTATTGTTTAATGATGTGTTTG
–817	CCTCTAATCAAACAATCTTAAAA
–718	AGAGGGTTTAAAGTTTTTTAAT
–258	GATTTTTTTGGGTTAATGGTA
–133	GGGTAGAGGAGGTGT
–64	CCCAATACCCCCAAA
–18	GGGAAGTTAGGGATT
	ELOVL2
–237	GGGTATTTTTGGGGTT
–292	GAAGTTTAATTGTAAGTAAGAT
–376	TTATTTTTTTTTAATGTGTGTTTTA
–429	AATAACTCTATTCCTACAAATT
–481	AGAGATTTTTGTTTAGGG
–6	ELOVL 5
–59	CCTAAACCCAAATTAACCCCTC
–134	GTAAAAAAATTAGAGTTTAGGG
–231	AGTATGATATAATTTATAGAGGAGA
–269	ACTAAAAAAAATCCACTTCATC
–633	AAATTAGAATTTTTAAGAAAAATAT

TSS, transcription start site.

### Analysis of mRNA expression

mRNA was isolated from PBMCs using Tri Reagent (Sigma, T9429). Complementary DNA was prepared and amplified by real-time RT PCR using SYBR Green Jumpstart Ready Mix (Sigma, S4438) as described previously [14]. The level of mRNA expression was quantified using commercially prepared primer pairs for FADS1 Hs_FADS1_1_SG QuantiTect primer assay, FADS2 (Hs_FADS2_1_SG QuantiTect primer assay), ELOVL 2 (Hs_ELOVL2_1_SG QuantiTect primer assay) (all from Qiagen). The assay conditions were as described by the manufacturer and were validated in house before use. ELVOL5 was quantified using bespoke primer sets (5′ to 3′ TATGAAGATTATCCGTGTC; 3′ to 5′ TGGCACCAAAATAAGAGT) (Eurofins Genomis, http://eurofinsgenomics.eu). Cycle parameters were 95°C for 2 minutes then 40 cycles of 95°C for 30 s, 55°C (cyclophilin, FADS1, FADS2, ELOVL2) or 60°C (ELOVL5) for 1 min and 72°C for 1 min. Samples were analysed in duplicate and the level of the individual transcripts was normalised to cyclophilin (Qiagen, Hs_PPIA_1_SG QuantiTect primer assay) by the standard curve method [15].

### Statistical analysis

There are no previous data sets of methylation levels of the genes of interest on which to base calculations of sample size. Therefore, retrospective analysis of statistical power was carried out for exemplar levels of methylation of 95%, 47% and 17% in FADS2. For these levels of methylation, 7 subjects per group would provide a statistical power of 80% to detect a difference of 10% with a probability of P<0.05. Male and female subjects were analyzed separately. The primary statistical analyses were as follows. Statistical comparisons of methylation level at baseline between male and female subjects were by Student’s unpaired t test. Comparison of end of treatment values with baseline values was by Student’s paired t test. The effect of treatment was determined by ANCOVA with supplement as a fixed factor and baseline methylation, age, BMI, proportion of lymphocytes and neutrophils as covariates with Bonferroni’s *post hoc* correction. The relationship between the level of methylation at individual CpG loci and that of the mRNA expression of the corresponding transcript was by Pearson’s correlation analysis.

## Results

### Subjects

There were no significant differences by one-way ANOVA in the characteristics of the subjects at baseline between the dietary supplementation groups in the Study 1 cohort (Table 1). However, the subjects in the Study 2 cohort were significantly younger (P<0.001) and had a lower BMI (P<0.001) than the subjects in the Study 1 cohort (Table 1). There were no significant differences in subject characteristics between males and females in either cohort.

### Methylation at baseline in the Study 1 cohort

**Figure 2:**
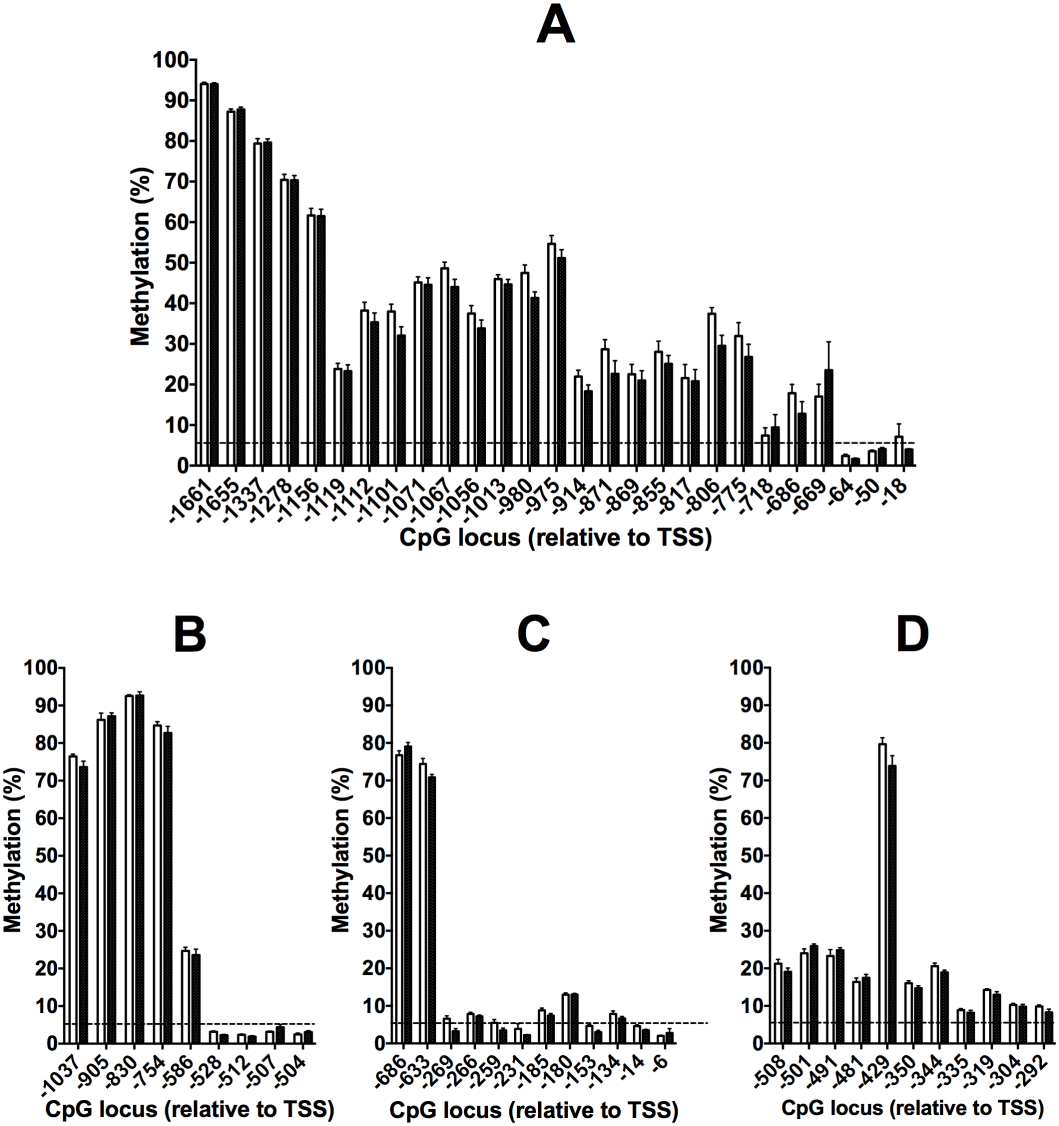
Values are mean ± SEM methylation of individual CpG loci at baseline in the 5′ regulatory regions of (A) FADS2, (B) FADS1, (C) ELOVL5 and (D) ELOVL2 in PBMCs from male (open bars) and female (closed bars) subjects in the Study 1 cohort. Numbers of subjects are listed in Table 1. Locations of individual CpG dinucleotides are relative to the transcription start site (TSS). Dotted horizontal line indicates the analytical limit of the assay.

Since the limit of detection was 5%, CpG loci that exhibited methylation of 5% or less were regarded as essentially unmethylated. This is consistent with previous reports [16]. The level of methylation of FADS2, FADS1 and ELOVL5 tended to be related inversely to distance from the transcription start site (TSS) (Figure 2 A–C). While this transition was gradual for FADS2, there appeared to be a sharp demarcation between a highly methylated (>70%) region distal to the TSS and the proximal region which was essentially unmethylated region in FADS1 and ELOVL5 which is consistent with the presence of CpG islands within the proximal promoters of FADS1 and ELOVL5 (Figure 1). Methylation of individual CpG loci in the 5′ regulatory region of ELOVL2 appeared to be essentially uniform with methylation levels between approximately 10% to 20%, with the exception of a CpG located at −429 bp from the TSS (CpG −429) which had a markedly greater level of methylation, approximately 80%, compared to the other CpG loci that were measured in this gene (Figure 2D).

### Effect of dietary supplementation with n-3 LCPUFA or OO on the methylation of individual CpG loci in the Study 1 cohort

**Figure 3:**
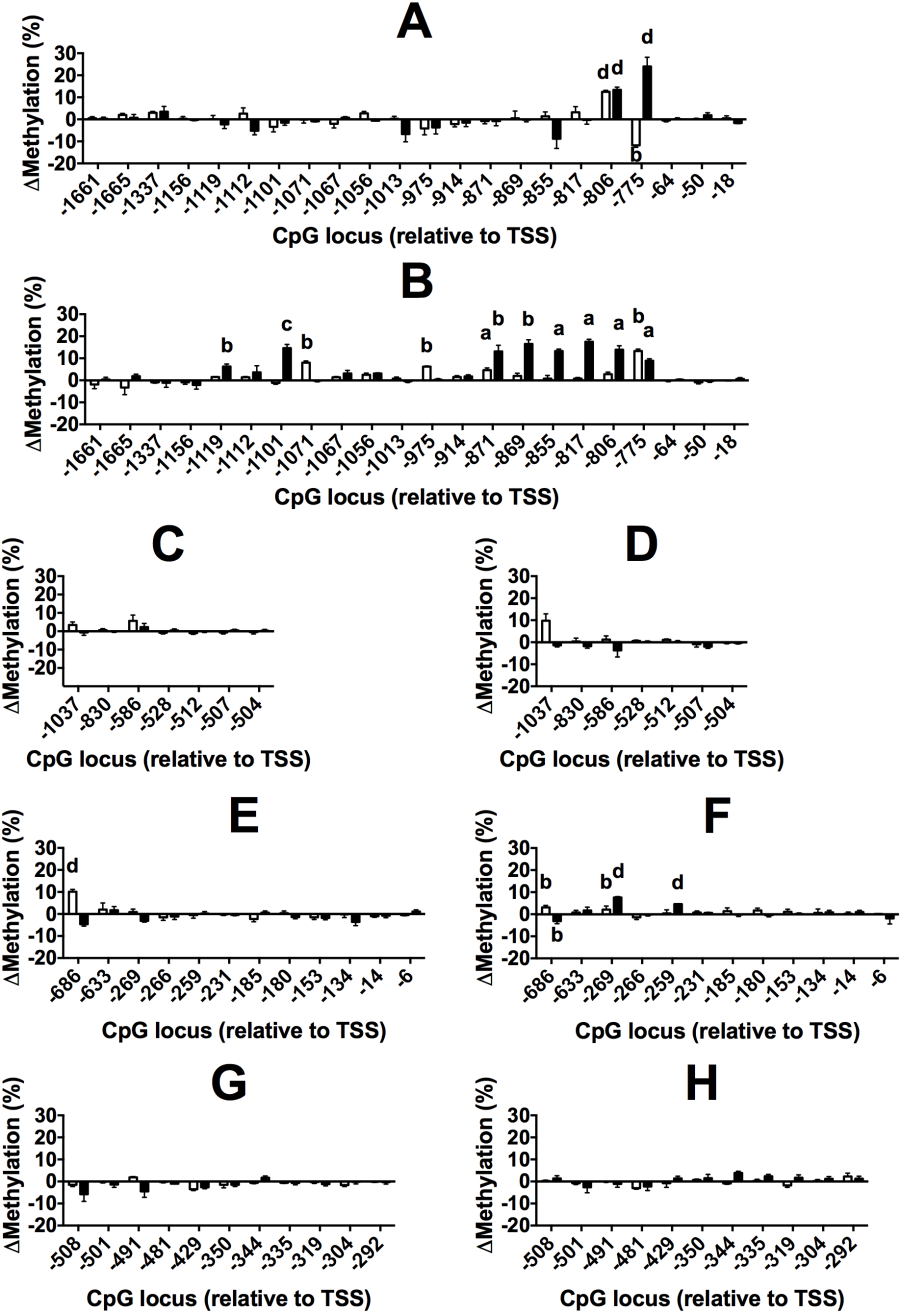
Values are mean ± SEM difference in methylation from baseline of individual CpG loci in the 5′ regulatory regions of (A, B) FADS2, (C, D) FADS1, (E, F) ELOVL5 and (G, H) ELOVL2 in PBMCs from male (A, C, E, G) and female (B, D, F, H) subjects in the Study 1 cohort who received n-3 LCPUFA (open bars) or OO (closed bars) supplements. Numbers of subjects are listed in Table 1. Locations of individual CpG dinucleotides are relative to the transcription start site (TSS). *Means that were significantly different by Student’s paired t test between baseline and end of intervention samples are indicated by ^a^P<0.05, ^b^P<0.01, ^c^P<0.001, ^d^P<0.0001.

Both OO and n-3 LCPUFA induced significant changes compared to baseline in the level of methylation of individual CpG loci in specific genes involved in PUFA metabolism in PBMCs in the Study 1 cohort that were contingent on sex. In males, n-3 LCPUFA increased methylation at CpG −806 (12.5%) in FADS2 compared to baseline, but decreased methylation at CpG−775 (11.8%) (Figure 3A). Supplementation with OO increased the level of methylation compared to baseline at both of these loci by 13% and 24.0%, respectively (Figure 3A). In females, n-3 LCPUFA supplementation induced increased methylation of FADS2 at CpGs −1071 (8.0%), −975 (6.2%), −871 (4.6%) and at CpG−775 (8.9%) compared to baseline (Figure 3B). Supplementation with OO increased methylation at CpGs −1119 (6.3%), −1101 (14.6%), −871 (13.1%), −869 (16.5%), −855 (13.3%), −817 (17.5%), −806 (13.9%) and CpG −775 (8.9%) compared to baseline (Figure 3B).

In males, supplementation with n-3 LCPUFA increased the level of methylation of CpG −686 (10.1%) compared to baseline in ELOVL5, but did not alter significantly the level of methylation of the other CpGs that were measured (Figure 2E). There was no significant effect of supplementation with OO on the methylation of CpG loci in ELOVL5 (Figure 2E). In females, n-3 LCPUFA supplementation increased the level of methylation of CpG −686 (3.1%) and CpG −269 (2.1%) in ELOVL5 compared to baseline (Figure 3F). Supplementation with OO decreased methylation of CpG −686 (3.1%), but increased methylation of CpG −269 (7.6%) and CpG −259 (4.6%) compared to baseline (Figure 2F). There was no significant effect of supplementation with either n-3 LCPUFA or OO on the level of methylation of any of the CpGs measured in FADS1 or in ELOVL2 in males or females (Figure 3C, D, G, H).

In males, the type of oil used in the dietary supplement induced differential methylation of CpG −775 (22%) in FADS2 (Table 4) and of CpG −686 (11%) in ELOVL5 (Table 5). In females, supplementation induced differential methylation of CpGs −1071 (8.5%), −975 (4.1%), −871 (8.2%), −869 (10.7%), −855 (10.0%), −817 (22.1%) and −775 (7.4%) in FADS2 (Table 4). The type of dietary oil supplement induced differential methylation at CpG −686 in males (11%) and in females (6.5%) in ELOVL5 (Table 5). There were no significant differences between the effects of the dietary supplements on the methylation of other CpG loci in FADS2 or ELOVL5, nor in the level of methylation of any other CpG loci measured in FADS1 or ELOVL 2 in males or females (Tables 4 and 5).

**Table 4:** Effect of supplementation with n-3 LCPUFA or olive oil on DNA methylation of FADS2 and FADS1 in the Study 1 cohort.

	Methylation of individual CpG loci (%)							
	Male				Female			
CpG	n-3 LCPUFA	OO	Adj n-3 LCPUFA	Adj OO	n-3 LCPUFA	OO	Adj n-3 LCPUFA	Adj OO
	FADS2							
–1661	95.0±1.6	93.6±1.2	95.1±0.5	93.4±0.6	92.4±4.4	94.0±1.5	93.7±1.5	93.7±1.2
–1665	88.7±1.1	88.7±1.8	88.8±0.4	88.7±0.5	84.6±2.2	89.5±1.3	84.5±2.3	89.6±2.2
–1337	83.7±3.5	81.6±3.2	82.8±0.8	81.3±0.8*	79.6±3.7	7.77±5.2	79.7±2.1	77.2±2.2
–1156	61.9±8.8	61.3±5.3	61.9±3.0	61.3±2.6	61.7±7.0	58.4±5.8	61.1±2.4	59.8±2.3
–1119	22.8±5.2	22.8±5.8	22.7±1.9	21.9±2.1	25.8±6.3	28.5±7.8	25.8±2.1	29.9±2.3
–1112	41.7±5.2	34.0±5.0	41.7±2.4	33.9±2.0	31.3±8.9	40.9±7.3	39.2±3.4	41.9±3.8
–1101	34.9±8.1	35.9±8.0	34.6±2.3	36.3±2.5	35.8±4.7	41.2±2.9	37.5±3.4	40.9±7.6
–1071	44.6±3.2	45.0±4.4	44.7±1.9	44.8±2.6	53.4±7.7	43.4±5.2	52.6±1.3	44.1±1.3*
–1067	46.7±4.7	49.7±8.1	46.5±2.0	49.6±2.7	47.2±6.3	46.3±7.3	48.3±4.0	47.6±3.6
–1056	36.9±7.3	39.0±8.4	40.14.4	39.1±3.5	35.7±5.9	37.4±7.1	35.6±4.2	37.1±3.4
–1013	46.1±3.0	39.4±8.8	46.2±3.0	39.4±2.6	44.8±4.5	43.7±4.7	44.9±1.8	44.1±1.8
–975	52.7±5.1	48.3±10.1	52.3±2.4	50.5±2.6	60.8±8.3	48.1±4.7	57.3±1.4	53.2±1.5*
–914	19.6±6.3	20.8±5.2	19.8±1.3	20.6±1.6	21.7±5.8	18.3±5.1	21.9±2.8	19.8±2.9
–871	25.4±5.8	30.3±4.4	27.9±1.6	29.3±1.7	30.6±7.2	33.1±8.3	27.2±3.0	35.4±2.8*
–869	21.9±5.4	24.0±6.4	22.6±2.7	23.6±3.6	27.4±5.9	33.1±4.0	23.7±1.5	34.4±1.9**
–855	27.6±4.9	22.1±9.8	29.1±2.8	20.8±3.9	29.2±4.7	35.6±4.4	28.7±1.0	38.7±1.4**
–817	24.1±5.4	21.2±4.9	23.7±4.3	22.4±3.6	17.7±11.8	31.5±5.6	14.8±4.9	36.9±5.7**
–806	50.0±2.3	50.8±2.0	50.0±0.9	50.9±1.0	38.0±9.2	37.7±5.7	37.8±1.5	51.6±4.0**
–775	23.6±7.8	45.8±4.3	23.6±7.7	45.8±4.36**	47.0±3.5	33.5±2.1	44.8±4.3	37.4±2.2**
–64	1.6±0.9	2.1±1.2	1.6±0.3	2.1±0.4	1.7±0.9	1.9±0.3	1.6±0.3	1.9±0.2
–50	3.8±0.9	5.7±3.4	3.7±0.6	5.7±0.8	3.6±0.5	3.5±0.9	3.6±0.3	3.5±0.3
–18	4.1±0.7	4.2±0.5	4.1±0.3	4.1±0.4	4.1±0.7	4.4±0.9	4.2±0.4	4.3±0.4
	FADS1							
–1037	76.3±4.3	78.2±4.3	77.9±2.8	76.6±2.2	74.8±2.0	75.4±5.2	72.7±2.0	69.7±1.9
–830	83.3±2.8	82.9±1.9	83.4±0.7	82.7±0.8	84.1±2.5	85.2±3.8	85.2±1.2	84.1±1.9
–586	26.5±5.7	28.1±5.1	27.2±2.0	27.8±1.9	23.0±5.1	23.3±3.6	23.7±1.6	22.7±2.3
–528	1.9±1.0	3.7±1.8	2.9±0.7	3.7±0.6	3.0±1.0	2.6±0.8	2.6±0.3	2.6±0.4
–512	1.0±1.2	2.2±1.7	1.8±0.6	1.8±0.3	2.8±0.5	2.8±0.7	2.9±0.2	2.7±0.3
–507	2.9±1.6	3.6±1.1	3.0±0.6	3.7±0.6	3.3±0.7	2.2±1.5	3.4±0.3	2.6±1.0
–504	2.0±2.1	2.6±1.2	2.2±0.5	2.6±0.5	3.0±0.9	2.46±1.4	3.1±0.3	2.8±.0.5

Values are mean or adjusted (Adj) mean ±SEM methylation of individual CpG loci at the end of the dietary intervention period in individuals who received either the n-3 LCPUFA or OO supplements. Numbers of subjects are indicated in Table 1. Comparisons between the level of methylation of individual CpG loci between supplementation groups at the end of the intervention were by ANCOVA. Means were adjusted for the level of methylation at baseline, age, BMI, and proportion of lymphocytes and neutrophils in blood, with Bonferroni’s *post hoc* correction. Adjusted means that were differed significantly are indicated by *P<0.05, **P<0.01, ***P<0.001, ****P<0.0001. Individual CpG dinucleotides are indicated by their position relative to the transcription start site (bp).

**Table 5:** Effect of supplementation with n-3 LCPUFA or olive oil on DNA methylation of ELOVL5 and 2 in the Study 1 cohort.

	Methylation of individual CpG loci (%)							
	Male				Female			
CpG	n-3 LCPUFA	OO	Adj n-3 LCPUFA	Adj OO	n-3 LCPUFA	OO	Adj n-3 LCPUFA	Adj OO
	ELOVL 5							
–686	83.4±3.4	76.4±2.5	85.0±1.1	74.0±2.8*	83.1±3.1	75.2±4.7	82.4±1.2	75.9±1.1**
–633	77.2±8.9	74.6±3.2	77.4±3.2	78.2±5.5	72.4±2.3	72.1±4.2	72.5±2.3	72.1±4.2
–269	5.6±1.2	5.1±1.8	5.8±0.6	4.3±0.6	9.8±1.2	4.8±1.5	4.7±0.7	5.9±1.3
–266	7.6±1.8	7.5±2.2	8.9±5.4	8.1±5.1	7.1±1.7	6.8±2.7	5.6±3.1	7.3±2.9
–259	5.3±0.8	5.1±0.7	5.3±0.3	4.9±0.3	4.5±1.9	6.6±0.8	5.1±0.9	8.2±1.3
–231	2.0±0.5	2.1±0.6	3.5±1.4	2.3±0.2	2.9±0.8	2.6±0.5	2.5±0.3	3.0±0.4
–185	9.8±2.6	8.2±1.1	8.0±0.5	9.4±0.7	8.1±1.0	9.9±2.1	9.3±0.8	8.1±0.9
–180	13.3±3.3	12.4±0.7	13.5±1.0	12.5±1.5	14.5±2.4	12.7±1.2	14.1±0.8	12.7±0.9
–153	2.8±1.5	3.5±1.0	2.5±0.4	3.4±0.6	3.9±0.9	3.3±1.5	3.8±0.4	2.9±0.5
–134	6.6±2.0	6.3±1.9	6.1±0.7	5.9±1.1	7.7±2.8	6.9±1.3	7.9±1.0	6.6±1.3
–14	3.6±0.9	3.7±2.0	3.6±0.4	3.8±0.5	3.7±1.5	4.6±1.2	3.7±0.5	4.6±0.5
–6	1.5±0.6	2.1±0.5	1.5±0.5	11.9±6.2	1.8±0.3	1.9±0.4	1.8±0.1	2.4±0.3
	ELOVL 2							
–508	18.4±3.2	16.8±7.3	19.4±2.3	16.8±2.1	17.3±6.4	20.6±5.5	15.7±2.2	21.9±2.3
–501	24.0±5.7	22.3±8.5	23.8±2.6	22.5±2.6	24.8±6.4	22.1±4.3	24.9±2.6	22.0±2.6
–491	21.4±4.5	22.4±5.3	21.8±2.4	22.0±2.0	24.1±6.2	23.8±6.2	23.8±2.3	24.12.1
–481	16.3±4.3	15.1±3.1	16.2±1.3	15.1±1.3	14.8±3.6	16.2±4.2	15.0±1.5	16.5±1.3
–429	77.5±6.9	74.4±4.2	78.0±2.0	75.1±2.4	76.4±4.1	71.1±4.9	76.5±2.2	71.9±2.5
–350	13.6±4.8	15.6±3.5	14.1±1.5	15.8±1.7	16.9±3.6	16.8±3.4	16.3±1.6	15.1±1.8
–344	20.1±5.0	22.1±3.7	20.0±1.2	21.9±1.4	19.3±2.7	21.5±2.3	18.8±1.2	21.1±1.3
–335	8.6±1.3	7.9±1.7	8.1±0.4	8.7±0.5	8.6±2.7	9.7±1.4	8.8±0.9	9.5±0.9
–319	14.1±1.9	12.6±4.9	14.1±1.1	13.1±1.4	12.1±6.7	14.9±2.6	10.8±1.7	14.7±1.7
–304	8.9±2.9	11.0±1.1	8.9±0.8	11.8±1.4	10.9±2.5	11.7±1.7	11.4±0.8	11.8±0.9
–292	9.9±4.3	9.8±1.6	9.8±0.8	11.2±0.8	10.2±1.5	10.3±2.7	10.0±0.8	10.9±0.9

Values are mean or adjusted (Adj) mean±SEM methylation of individual CpG loci at the end of the dietary intervention period in individuals who received either the n-3 LCPUFA or OO supplements. Numbers of subjects are indicated in Table 1. Comparisons between the level of methylation of individual CpG loci between supplementation groups at the end of the intervention were by ANCOVA. Means were adjusted for the level of methylation at baseline, age, BMI, and proportion of lymphocytes and neutrophils in blood, with Bonferroni’s *post hoc* correction. Adjusted means that were differed significantly are indicated by *P<0.05, **P<0.01, ***P<0.001, ****P<0.0001. Individual CpG dinucleotides are indicated by their position relative to the transcription start site (bp).

### Effect of dietary supplementation with n-3 LCPUFA on the methylation of individual CpG loci in the Study 2 cohort

**Figure 4:**
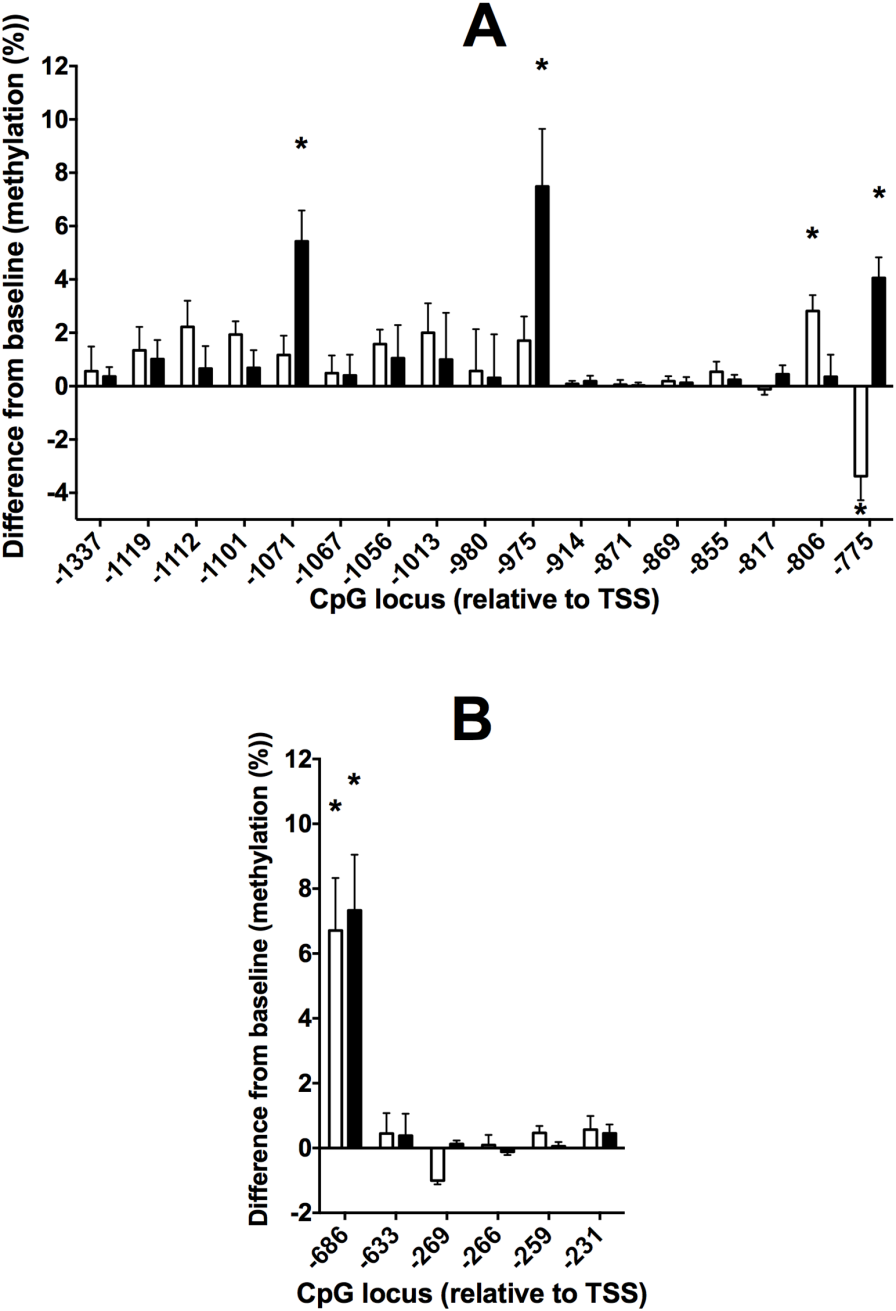
Values are mean ± SEM difference in the methylation of individual CpG loci from baseline in the 5′ regulatory regions of (A) FADS2 and (B) ELOVL5 in PBMCs from male (open bars) and female (closed bars) in the Study 1 cohort who consumed a n-3 LCPUFA supplement. Numbers of subjects are listed in Table 1. Locations of individual CpG dinucleotides are relative to the transcription start site (TSS). *Means that were significantly different (P<0.05) by Student’s paired t test between baseline and end of intervention samples.

We investigated whether the changes in methylation induced by supplementation with n-3 LCPUFA in the Study 1 cohort could be replicated in a second cohort of health individuals. For FADS2, n-3 LCPUFA supplementation in the Study 2 cohort also induced an increase in methylation at CpGs −803 and −775 in males and in CpGs −1071, −975 and −775 in females (Figure 4A) to those induced in the UWA cohort (Figure 2 A, E). However, increased methylation at CpG −871 in females in the UWA cohort was not replicated in the Study 2 cohort (Figure 4A). For ELOVL5, the level of methylation of CpG −686 was increased compared to baseline in both the Study 1 and Study 2 cohorts (Figure 4B). However, the level of methylation of CpG −269, which was increased compared to baseline in females who received the n-3 LCPUFA supplement in the Study 1 cohort, was unchanged in females in the Study 2 cohort. CpGs in FADS2 or ELOVL5 that were measured in the same amplicons as those that showed altered methylation, but which did not exhibit a change in methylation following n-3 LCPUFA supplementation in the Study 1 cohort, also did not show altered methylation in the Study 2 cohort (Figure 4).

### The relationship between the methylation of individual CpG loci and the mRNA level of the corresponding transcripts

**Figure 5:**
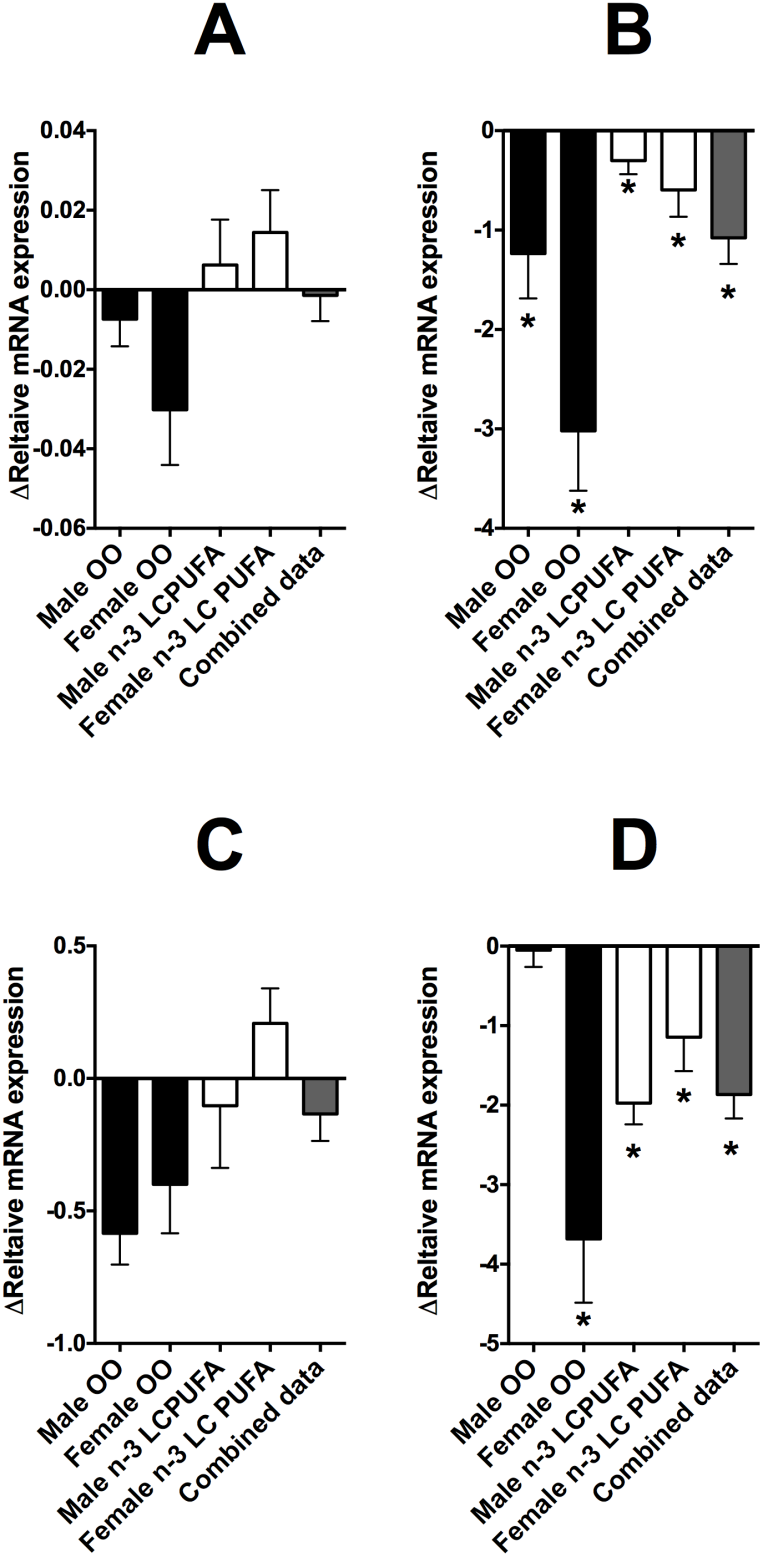
Change in relative mRNA expression of (A) FADS1, (B) FADS2, (C) ELOVL2 and (D) ELOVL5 in peripheral blood mononuclear cells from Study 1. *Means at the end of the end of the study that were significantly different (P<0.05) compared to baseline assessed by Student’s paired t test. Combined data refers to the overall change in mRNA expression irrespective of sex or supplement (these data were used to test the statistical association with the methylation status of the respective genes).

**Figure 6:**
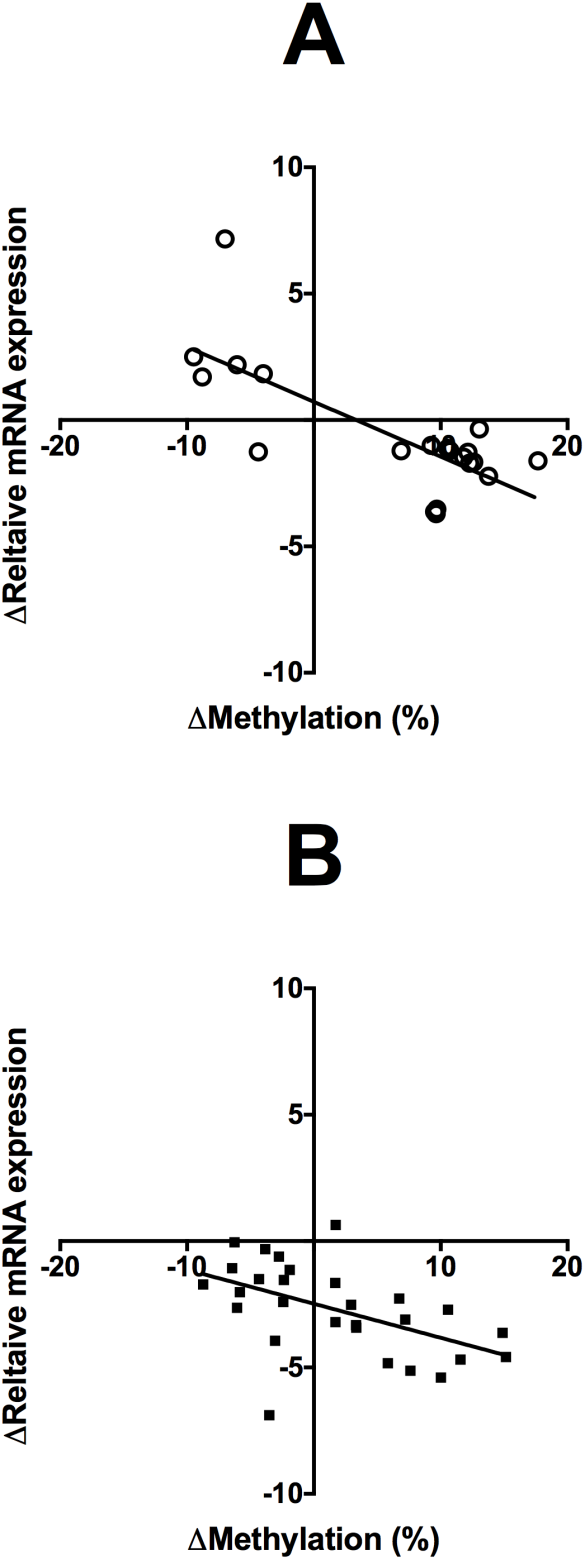
Change in relative mRNA expression compared to change in the methylation status of (A) FADS2 CpG −775 and (B) ELOVL5 CpG −686 irrespective of subject sex or supplementation group in peripheral blood mononuclear cells from Study 1. The corresponding correlation efficients are shown in Table 6.

The level of methylation of specific CpG loci in FADS2 and ELOVL5, but not FADS1 nor ELOVL2, in the Study 1 cohort were associated significantly the level of the mRNA transcript in samples collected at the end of the study irrespective of subject sex or dietary supplement. There were no significant differences in FADS1 or ELOVL2 mRNA expression between baseline and end of study samples (Figure 5 A, C). However, the mRNA expression of FADS2 was reduced significantly in all dietary groups, while ELOVL5 expression was reduced in females who received the OO supplement and in males who received with the OO or n-3 LCPUFA supplement at the end of the study compared to baseline (Figure 5 B, D). The CpGs at −119, −871, −869, −806 and −775, which showed altered methylation in response to dietary supplementation, were associated negatively with the level of the FADS2 transcript (Table 6). The CpGs −686 and −259 in ELOVL5, which showed altered methylation in response to dietary supplementation, were associated negatively with the level of the ELOVL5 transcript (Table 6). These associations are illustrated for FADS2 CpG −775 and ELOVL 5 CpG −686 in Figure 6.

**Table 6:** The relationship between the methylation status of individual CpG loci at the end of the intervention study and expression of the corresponding mRNA in the Study 1 cohort.

Pearson’s correlations											
FADS2			FADS1			ELOVL 5			ELOVL 2		
CpG	r	p	CpG	r	p	CpG	r	p	CpG	r	p
–1661	–0.05		–1037	–0.06		–686	–0.32	0.009	–508	0.3	
–1665	–0.06		–830	–0.5		–633	0.04		–501	–0.44	
–1337	0.03		–586	0.09		–269	–0.11		–491	0.20	
–1156	0.21		–528	–0.1		–266	–0.15		–481	0.1	
–1119	–0.57	0.009	–512	–0.24		–259	–0.18	0.038	–429	–0.25	
–1112	0.15		–507	0.05		–231	–0.22		–350	–0.08	
–1101	0.18		–504	–0.05		–185	–0.1		–344	0.05	
–1071	–0.12					–180	–0.37		–335	0.24	
–1067	0.11					–153	–0.1		–319	0.08	
–1056	0.17					–134	0.0		–304	0.29	
–1013	0.14					–14	–0.07		–292	–0.12	
–975	0.11					–6	–0.08				
–914	0.01										
–871	–0.17	0.047									
–869	–0.31	0.017									
–855	0.08										
–817	0.12										
–806	–0.52	0.007									
–775	–0.21	0.043									
–64	–0.02										
–50	–0.06										
–18	0.02										

Values are Person’s correlation coefficients and probabilities for the relationship between methylation of individual CpG loci and the level of the corresponding mRNA transcript at the end of the intervention study. Numbers of subjects are indicated in Table 1.

## Discussion

The findings of this study show for the first time that modest dietary supplementation with either n-9 monounsaturated fatty acids or n-3 LCPUFA induces altered DNA methylation in specific genes involved in LCPUFA metabolism in leukocytes from adults that differed between the type of supplement and between sexes.

The methylation profile of CpGs within the 5′ regulatory region of the four key genes involved in LCPUFA biosynthesis has not been reported previously in humans. FADS2 was characterised by a gradual decline in the level of methylation of individual CpG loci 90% to 20% with decreasing distance from the transcription start site. Previous reports in rodents have shown that the level of methylation of the Fads2 promoter in mice was less than 10% [8], [17], while in rats the level of methylation was between 30% and 90%, increasing with distance from the transcription start site [10], [11]. This implies that the epigenetic regulation of this gene may be more similar in humans to rats, than to mice, which may have implications for understanding the epigenetic regulation of FADS2 in these species. However, since FADS2 appears to have multiple TSS (http://www.ensembl.org/Homo_sapiens/Gene/Summary?db=core;g=ENSG00000134824;r=11∶61560452–61634826), detailed promoter mapping in the different species would be necessary to establish differential species-specific regulation of this gene. The level of methylation of some CpG loci in the 5′ regulatory region of FADS2 has been reported previously in HepG2 cells [18]. Despite prolonged passage of this hepatocellular carcinoma cell line in culture, the methylation levels of the CpG loci that were measured were comparable to those reported here, with the exception of CpG −1661, which had approximately 30% lower methylation in HepG2 cells [18]. Whether this difference in methylation reflects an effect of prolonged adaptation to *in vitro* culture or differences in cell type between leukocytes and hepatocellular carcinoma cells cannot be determined from these findings. However, together these observations suggest that the epigenetic regulation of the FADS2 gene may be similar in the two distinct cell types and that HepG2 cells may be a suitable model of the epigenetic regulation of FADS2 in humans.

FADS1 and ELOVL5 showed a marked demarcation between a highly methylated region distal to the transcription start site and an essentially unmethylated proximal region. ELOVL2 showed a similar level of methylation, about 20% across the region that was measured with the exception of CpG −429 that showed markedly higher methylation. However, the role of this CpG locus in the regulation of ELOVL2 and the effect of differential methylation of this site on the transcription of the gene is not known.

There is substantial evidence that women have higher DHA status [19] and capacity for DHA synthesis [20], [21] than men. This sex difference has also been shown in rodents to be accompanied by differential mRNA expression of Fads2 and Fads1 in the liver [22], [23]. In the present study, we found no differences between men and women in the methylation status of individual CpG loci in any of the gene regions that were investigated. This suggests that differential methylation of FADS1 or 2, or of ELOVL 2 or 5 may not contribute significantly to the sex difference in DHA status. Previous studies have shown that supplementation of pregnant rodents with different types and amounts of dietary fats induced hypermethylation of specific CpG loci in the Fads2, but not Fads1, promoter in the liver and aortae of the offspring [10], [11]. The nature of such changes differed according to the type and amount of fat in the maternal diet. Similarly, feeding adult rats a diet enriched in fish oil induced hypermethylation of specific CpG loci in the Fads2 promoter which was reversed by withdrawal of the high n-3 LCPUFA diet [10]. Feeding adult male mice a high fat diet induced hypermethylation of specific CpG loci in stearoyl-CoA desaturase [24]. Treatment of cells in culture with EPA has also been shown to induce altered methylation of sCCAAT/enhancer binding protein-β [25]. To our knowledge there have been relatively few studies of the effect of dietary fat on DNA methylation in humans. Consuming a high fat diet for 5 days induced altered methylation of 7,909 CpG loci in 658 genes in skeletal muscle from young men [26]. Feeding 400 mg DHA per day to pregnant women from mid gestation to term induced a small increase (about 1%) in the methylation status of LINE-1 sequences in leukocytes from umbilical cord blood [27]. However, the nature of the latter study design may limit the interpretation of these findings [28]. The current findings show that consuming supplements enriched in n-9 monounsaturated fatty acids or n-3 LCPUFA induces differential changes in the methylation of individual CpG loci in two out of the four genes that were investigated. Such differential effects are consistent with previous findings in rodents [11]. Furthermore, the pattern of induced changes in DNA methylation differed between men and women such that in terms of the number of CpG loci that were affected, the effect of the dietary supplements was greater in women than in men in both FADS2 and ELOVL5.

The effects of supplementation with n-3 LCPUFA that were observed in the patients with chronic kidney disease were fairly well replicated in the cohort of healthy individuals in terms of the number and location of the CpGs that were changed in both FADS2 and ELOVL5, and the direction of the effect on these genes. There were two exceptions, CpG −871 in FADS2 and CpG −269 in ELOVL5 showed increased methylation (approximately 5%) in women in the Study 1 cohort who consumed the n-3 LCPUFA supplement, but were unchanged in the Study 2 cohort. However, overall these findings suggest that effect of supplementation with n-3 LCPUFA on DNA methylation of FADS2 and ELOVL5 was not affected significantly by differences in the fatty acid composition of the n-3 LCPUFA preparations, or the age, BMI, health status or geographical location of the subjects. Since the changes induced in the methylation of FADS2 and ELOVL5 were similar after either 8 or 12 weeks supplementation, these findings also suggest that the effects of increased intakes of n-3 LCPUFA had reached a maximum response by 2 months. CpGs in FADS2 and ELOVL that were proximal to those that showed altered methylation but were unaffected by the dietary supplement in the Study 1 cohort, were also unaffected in the Study 2 cohort. This confirms the specificity of the effects of the different fatty acid supplements.

The methylation status of specific CpG loci in Fads2 has been shown previously in rats to directly influence the level of transcription [11]. We investigated whether the level of methylation of individual CpG loci in the four genes of interest was associated with the level of the corresponding transcripts. We found that the methylation status of four CpG loci in FADS2 and one CpG loci in ELOV5, which showed induced changes in methylation following dietary supplementation, were negatively associated with the level of the FADS2 and ELOVL5 transcripts, respectively. Although this does not provide a direct functional assessment of the effect of altered methylation at these loci on the level of transcription, such associations are consistent with increasing DNA methylation inducing a lower level of transcription. Increased total dietary fat intake [6], [7], or supplementation with olive oil [29] or n-3 LCPUFA [30] has been shown to decrease the conversion of 18 carbon essential fatty acids to their longer chain metabolites. The present findings suggest that increased intakes of n-3 LCPUFA or olive oil may reduce PUFA biosynthesis via changes in the epigenetic regulation of FADS2 and ELOVL5 that affect gene transcription, possibly in addition to any effect of product inhibition. Since olive oil is not a pure preparation of fatty acids, it cannot be assumed that the changes in DNA methylation were due to the lipid content of the oil since other biologically active compounds are present such as polyphenols [31]. Because D6d is the rate-limiting enzyme in PUFA biosynthesis, lower transcription of FADS2 would tend to down-regulate the activity of the whole pathway. However, lower transcription of ELOVL5 may reduce conversion of dihomo-γ-linolenic acid to archidonic acid and EPA to DPAn-3. If so, one possible consequence could be to increase the synthesis of substrates for the synthesis of prostaglandin (PG) E_1_ and PGE3, relative to PGE_2_.

The nature of this study design has some limitations. Although retrospective analysis suggested that the study was adequately powered to detect differences of 10%, changes in methylation of less than10% need to be considered cautiously. Replication of the study in a larger prospective cohort of healthy individuals, preferably with a crossover rather than parallel design, would be desirable to substantiate the current findings. It would also be desirable to test the dietary supplements at more than one dose. Because cell differentiation involves differential changes in the methylation of individual genes, it is possible that variations in cell populations may have contributed to the variation in the level of methylation of individual CpG loci, although differences in the proportions of individual cell populations were taken into account in the analysis of the samples from the Study 1 cohort. Furthermore, the similarities in the findings from the Study 2 cohort, which were based on DNA isolated from whole blood without correction for leukocyte sub-types, to those from the Study 1 cohort supports the suggestion that variation in cell populations does not significantly affect the methylation of these genes. Finally, although there were significant associations between the methylation status of individual CpG loci and the level of the corresponding mRNA transcripts, the effect of variation in DNA methylation of these genes on their transcription and on PUFA biosynthesis remain to be demonstrated directly.

Overall, the findings of this study show that dietary supplementation of adult humans with modest amounts of n-3LCPUFA or olive oil can induce selective changes in the methylation status of individual CpG loci in specific genes, which is contingent on the sex of the subject and the nature of the supplement. Such findings may have implications for understanding the mechanisms the underlie the health benefits associated with higher consumption of fish oil [32], [33] or olive oil [34]. Furthermore, altered DNA methylation has been implicated as a causal process in a number of non-communicable diseases [4], [5]. One challenge in the design of therapeutic strategies to ameliorate or reverse altered patterns of DNA methylation in disease states is targeting the intervention to specific CpG loci [35]. One possible implication of the current findings is that supplementation with specific fatty acids or combinations of fatty acids may provide a means of delivering health benefits by inducing selective modification of the methylation status of specific CpG loci.
